# Hyperplastic Dental Follicle: A Case Report and Literature Review

**DOI:** 10.1155/2014/251892

**Published:** 2014-10-08

**Authors:** Ligia Buloto Schmitd, Diego Maurício Bravo-Calderón, Cleverson Teixeira Soares, Denise Tostes Oliveira

**Affiliations:** ^1^Pathology Division, Department of Stomatology, Bauru School of Dentistry, University of São Paulo, Alameda Octávio Pinheiro Brisolla 9-75, 17012-901 Bauru, SP, Brazil; ^2^ESFA Dental School, Rua Bernardino Monteiro 700, 29650-000 Santa Teresa, ES, Brazil; ^3^Lauro de Souza Lima Institute, Rodovia Comandante João Ribeiro de Barros, Km 225/226, 17034-971 Bauru, SP, Brazil

## Abstract

Hyperplastic dental follicle is an odontogenic hamartomatous lesion associated with delayed or tooth eruption failure in young patients. The occurrence of this pericoronal dental lesion may be single or multiple and it seems to be more frequent than literature has reported. We present a literature review focusing on the etiopathogenesis and clinicopathological features of this hamartomatous lesion in young patients. In addition, we reported a case of hyperplastic dental follicle causing delayed tooth eruption of 14-year-old male patient. Microscopic analyses based on routine staining and immunohistochemistry were used to discuss the cells found in pericoronal follicle. This paper reinforces the importance of association between clinical history and radiographic features with microscopic pericoronal follicle examination for diagnosis of this hamartomatous lesion.

## 1. Introduction

Hyperplastic dental follicle has been described as odontogenic hamartomatous lesion that occurs in pericoronal tissues of the unerupted tooth [1, 2]. Although its occurrence can be found in any age, most cases in the literature affected young individuals [3–7], involving permanent first and second molars [3, 7–9].

The radiographic appearance of hyperplastic dental follicle is characterized by well-circumscribed radiolucent area with sclerotic borders surrounding the crown of an unerupted tooth, frequently mimicking dentigerous cyst [4, 10]. Delayed or tooth eruption failure has been associated with this hamartomatous lesion. Microscopically, the hyperplastic dental follicle consists of fibrous connective tissue containing odontogenic epithelium, multinucleated giant cells, and calcification foci [3, 8, 11].

Recently, some authors described the occurrence of multiple calcifying hyperplastic dental follicles associated with multiple unerupted teeth affecting young male patients [5, 12, 13] and they suggested that this condition should be considered a distinct pathology [12, 13].

The occurrence of hamartomas from odontogenic origin seems to be more frequent than that which has been reported in the literature [14–16]. Furthermore, the diagnosis of hyperplastic dental follicle is important in order to distinguish this condition from other odontogenic tumors that present different pathogenesis and recurrence potential [8].

We use this case of hyperplastic dental follicle as an opportunity to review the literature focusing on the etiopathogenesis and clinicopathological features of this hamartomatous lesion in young patient.

## 2. Case Presentation

**Figure 1:**
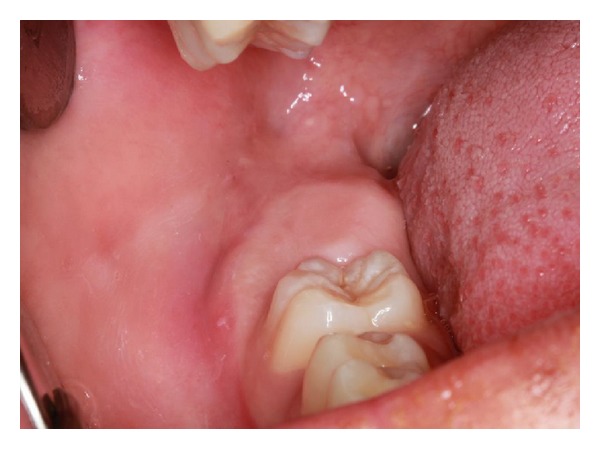
Enlargement of gingival area associated with unerupted right mandibular second molar.

A 14-year-old black male presented to the Dental Clinic at Escola Superior São Francisco de Assis, Espírito Santo, Brazil, with the chief complaint of absence of a molar tooth in the inferior dental arch. His medical history was unremarkable. Clinical examination showed enlargement of the gingival region of unerupted right mandibular second molar (Figure 1).

**Figure 2:**
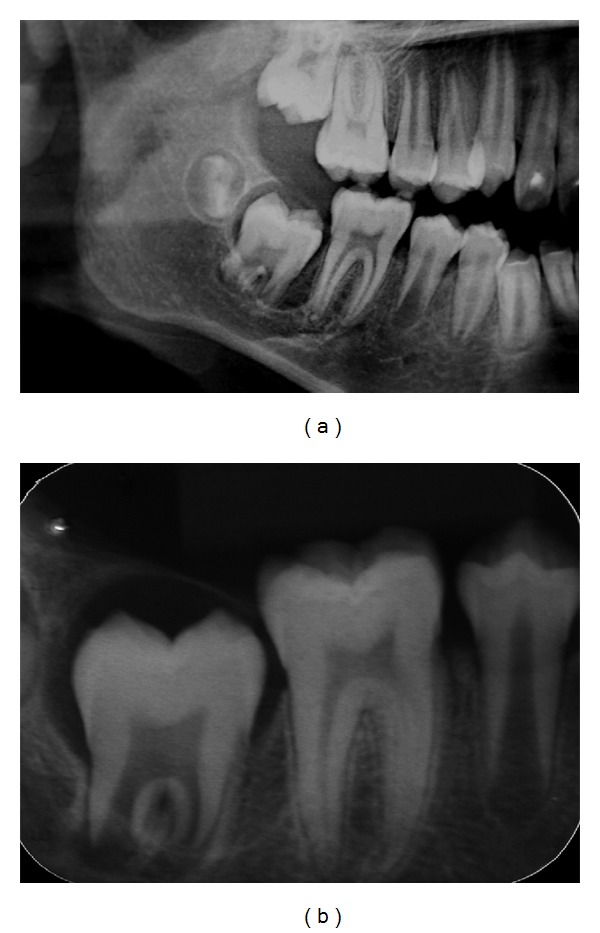
Radiographic aspect of the unerupted teeth. (a) Sectioned panoramic radiography: radiolucent well-defined area surrounding the crown of tooth 47, extending to apical region in the anterior area. (b) Periapical radiography: delicate sclerotic border, normal enamel and radicular formation, and absence of visible calcifications in pericoronal space.

**Figure 3:**
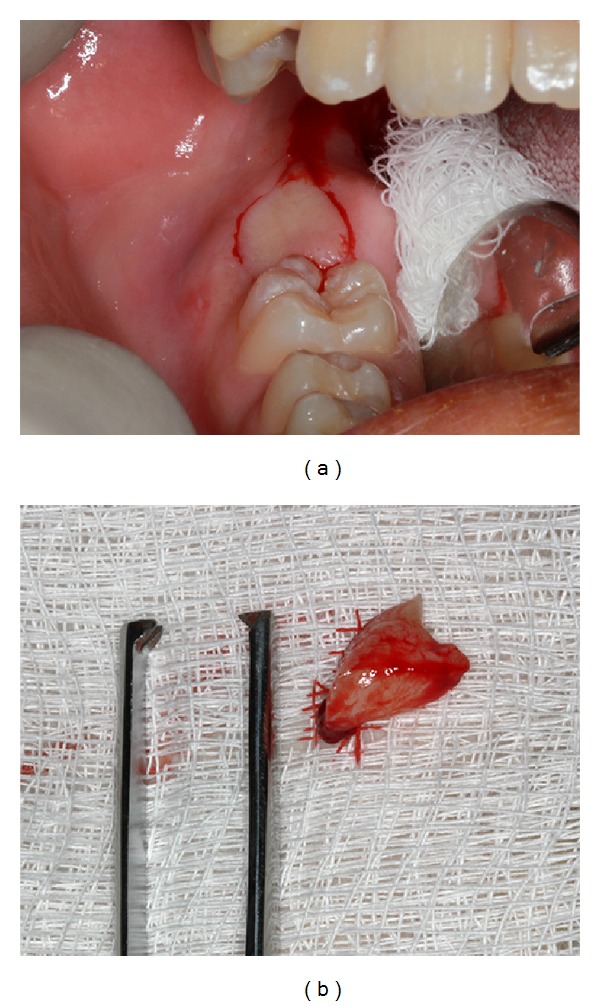
(a) Incisional biopsy of the lesion. (b) Fresh fragment of gingival and follicular tissues after removal.

Radiographic examination revealed a radiolucent well-delimited area surrounding the crown of the unerupted tooth, with one-third of root formed and calcified. In addition, periapical radiography showed that dental follicle was limited by thin sclerotic border with absence of visible calcifications. The radicular and enamel formation of the tooth were normal (Figure 2). The clinical diagnosis was dentigerous cyst. No other radiologic abnormalities involving teeth were observed. Surgical marsupialization of the lesion was performed under local anaesthesia exposing the dental crown and no fluid accumulation was observed. The pericoronal follicle was submitted to the Laboratory of Pathology of Bauru School of Dentistry, University of São Paulo, for histopathological analysis (Figure 3).

**Figure 4:**
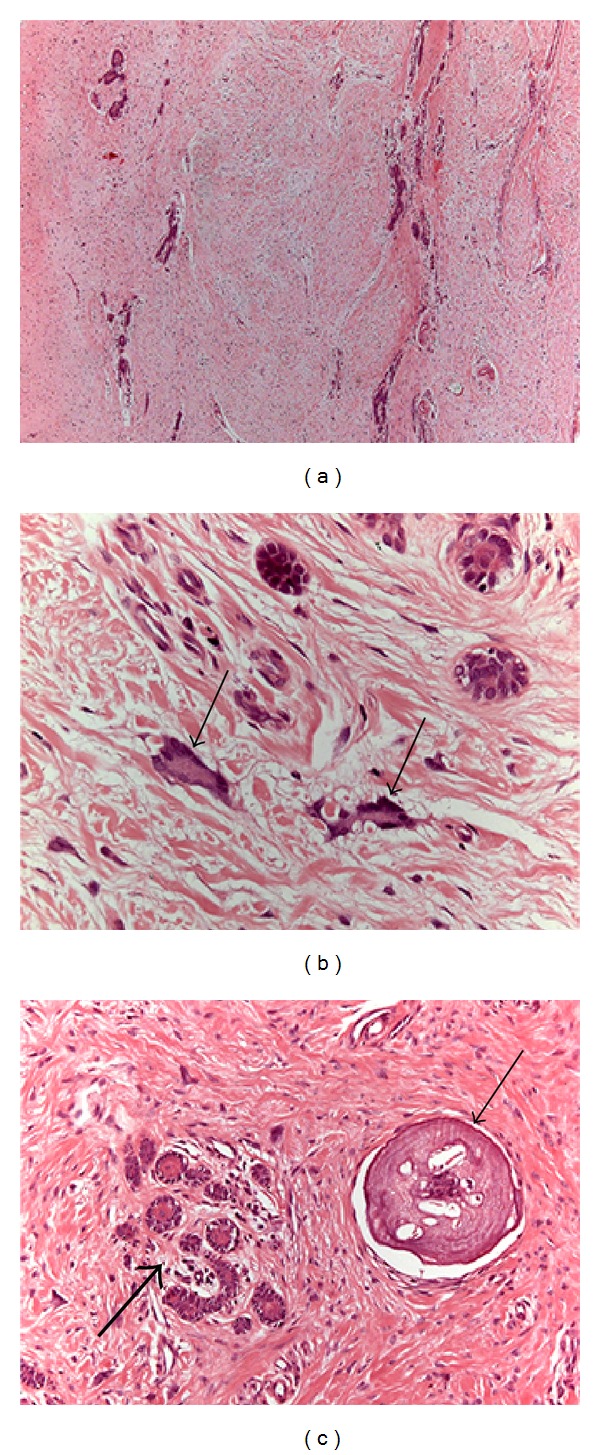
Histological aspect of the follicle: (a) pericoronal dental tissue with dense connective tissue; (b) multinucleated giant cells (arrows); (c) islands of odontogenic epithelium (large arrow) and calcified focus (small arrow) (H&E; (a) = 50x, (b) = 400x, and (c) = 200x).

**Figure 5:**
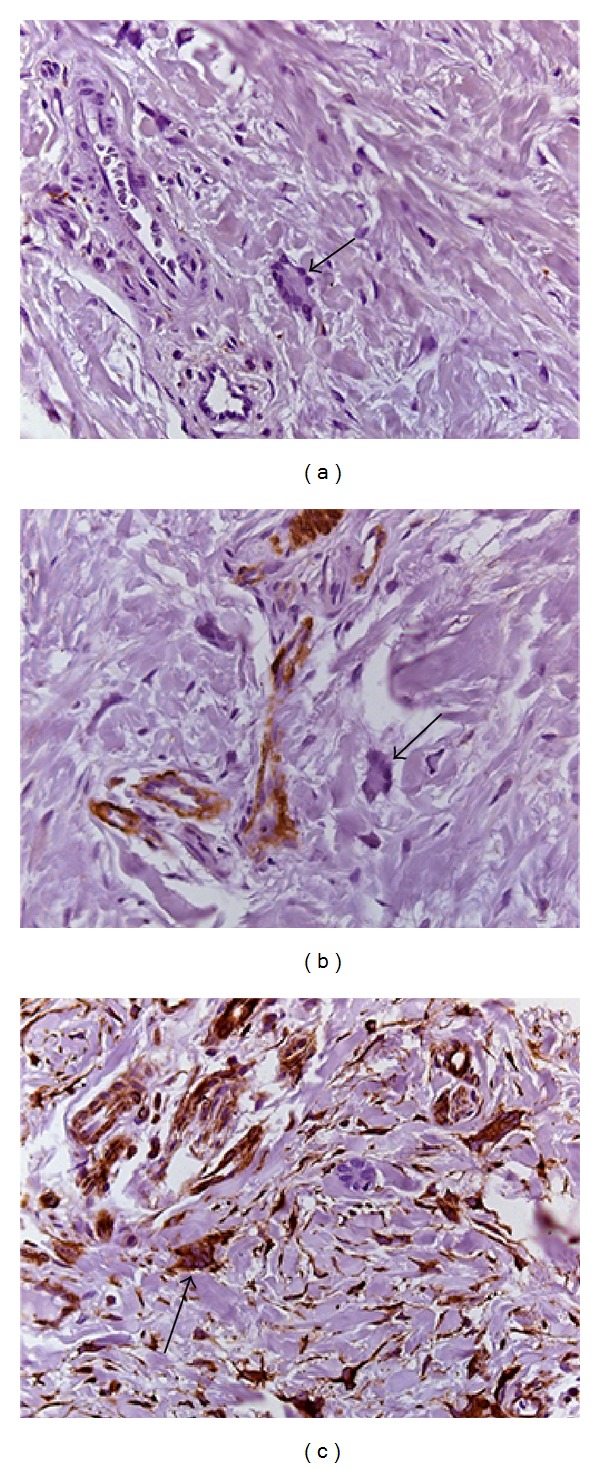
Microscopic immunohistochemical features: (a) the giant multinucleated cells showed negative reactivity for antibody anti-CD-68 (arrow) indicating no monocyte origin; (b) the blood vessels were positive for anti-HHF-35 but the giant multinucleated cells were negative for this antibody (arrow); (c) strong vimentin immunopositivity of the giant multinucleated cells was observed indicating mesenchymal origin (arrow) ((a) = 400x, (b) = 400x, and (c) = 400x).

Microscopic examination revealed noninflamed fibrous connective tissue with dense collagen, fusiform cells, and giant multinuclear cells (Figures 4(a) and 4(b)). Odontogenic epithelial islands were scattered randomly and surrounded by calcification focus (Figure 4(c)). Some of these epithelial islands presented squamous metaplasia. Reduced enamel epithelium was not identified. The immunohistochemical analysis showed that the multinucleated giant cells were negative for CD-68, HHF-35 and strongly positive for vimentin. The blood vessels were positive for HHF-35 and the collagen fibers for vimentin (Figure 5). Based on clinical, radiological, and microscopic examination, the diagnosis established was hyperplastic dental follicle. The patient is under clinical control and the tooth is in eruption process.

## 3. Review of the Literature

We reviewed the English literature for hyperplastic dental follicle occurring in young patients (under 21 years old) and the following inclusion criteria were used: (1) patients under 21 years old, (2) complete description of the teeth involved, (3) absence of systemic diseases, and (4) microscopic analysis of the dental follicle. Based on these criteria, we selected 13 reports, including the present case, and the data are summarized in Table 1.

## 4. Discussion 

Over the past decades, some clinical reports had explored the characteristics of the hyperplastic dental follicle associated with delayed or tooth eruption failure, in an attempt to categorize it as a pathological distinct entity [8, 11]. Radiographically, normal pericoronal follicle is considered to be in the range of 2-3 mm [16]. Although the radiographic evidence of a radiolucency around the crown of an unerupted tooth of no more than 5 mm in width is strongly suggestive of dentigerous cyst or odontogenic tumors, the hyperplastic dental follicle should be considered in the clinical diagnosis [1, 17, 18].

The etiopathogeny of hyperplastic dental follicle is unclear and in some reported cases the teeth affected presented defective enamel formation such as amelogenesis imperfecta [5, 7, 10] or enamel dysplasia [9].

According to our English review, the patients' age range varied from 5 years to 19 years old (Table 1) and the relationship between female and male was 1 : 1.4, showing higher occurrence of hyperplastic dental follicle in young male than in female gender.

Of the 18 patients with hyperplastic dental follicles found in the literature and that matched our inclusion criteria (Table 1), most of them involved multiple teeth (14 cases reported). Only 4 patients under 21 years presented with single affected tooth, including our case reported. Regarding the multiple cases, the number of involved teeth ranged from 4 to 16 different teeth. Two authors reported patients with hyperplastic dental follicles affecting two distinct teeth in the same individual (first and second mandibular molars) and it was not considered multiple occurrence [3, 8].

When the characteristics of enamel mineralization were analyzed we verified that the teeth crowns were normal in 14 patients with hyperplastic dental follicle and 4 patients presented with amelogenesis imperfecta or enamel dysplasia (Table 1). Furthermore, it is important to reinforce that there is no relationship between multiple hyperplastic dental follicle and genetic syndromes involving multiple unerupted teeth such as Gardner syndrome and cleidocranial dysplasia [19].

Microscopic findings in hyperplastic dental follicle include the presence of fibrous connective tissue, wavy collagen fibers, strands and islands of odontogenic epithelium, multinucleated giant cells, and varying sizes of basophilic mineralized areas presenting round cementum-like or psammomatous calcifications [12, 19]. In Table 1, we described all cases of hyperplastic dental follicle with calcification foci in patients under 21 years. These calcified areas varied in size, amount, and microscopic appearance, some of them resembling woven bone, osteodentin, and cementum while others presenting psammomatous calcification and Liesegang ring-like structures [5, 9, 12, 13, 17]. The presence of morphologically distinct calcifications associated with nests of odontogenic epithelium is frequently observed in normal pericoronal dental tissues or in odontogenic cyst and tumors [20, 21]. Moreover, according to our review (Table 1), calcification areas seem to be a common finding in hyperplastic dental follicle of the young individuals.

The histopathological diagnosis of hyperplastic dental follicle is based on hematoxylin eosin routine staining. Although immunohistochemistry is not necessary to establish the final diagnosis, in the present case, the immunoprofile of the giant cells was investigated. The hyperplastic dental follicle showed typical characteristics such as positive reactivity of the collagen for vimentin and of the blood vessels for HHF-35. Furthermore, the multinucleated giant cells were negative for CD-68, HHF-35 and strongly positive for vimentin, confirming the mesenchymal origin of these cells. According to previous study [11], the multinucleated giant cells in hyperplastic dental follicle are fibroblasts and seem to be associated with the production of myxoid matrix. It is important to reinforce that the presence of stellate and giant fibroblasts is commonly detected in gingival tissue of young individuals [2].

Since 1980, when Gardner [1] first described the hyperplastic dental follicle, there are difficulties regarding the pathological differentiation of this hamartomatous lesion with other odontogenic tumors, particularly with central odontogenic fibroma. Both lesions present similar clinical and histopathological characteristics and the distinction may be challenging [1, 17, 21, 22]. It has been considered that hamartomatous lesion such as hyperplastic dental follicle presents a less aggressive behavior than tumors as odontogenic fibroma and that the clinical outcome may be an important tool for diagnosis [8, 21].

The mechanism by which some follicles become hyperplastic and cause retarded eruption or impaction of teeth is not completely known. However, it has been suggested that the presence of hamartomatous pericoronal areas as observed in our case reported may induce active tissue remodeling and result in fibrosis [2, 11]. On the other hand, Kim et al. [23] confirmed that the turnover of extracellular matrix is negatively affected by downregulation of metalloproteinases in hyperplastic dental follicle when compared to normal pericoronal follicle, suggesting this factor as the responsible for abnormal tooth eruption.

Our case reported and other clinical cases reviewed reinforce the need to perform a careful radiograph examination in young patients with single hyperplastic dental follicle in order to investigate the possible multiple occurrence of this lesion affecting unerupted teeth. Whenever possible, follicles causing delayed eruption should be removed in order to release the teeth from impaction and the routine microscopic examination should be performed in order to differentiate the hamartomatous follicle from other neoplastic odontogenic tumors [14].

Based on present case and in our review, the diagnosis of hyperplastic dental follicle in young patients should be established when a clinical history of delayed tooth eruption exhibits radiographic image of enlarged pericoronal space in association with distinct microscopic features. So, the clinicians should be aware of the existence of this pathology in young individuals and systematically send removed pericoronal follicles to microscopic analysis.

In conclusion, we presented a literature review and a case of hyperplastic dental follicle causing delayed tooth eruption in a young patient, emphasizing the importance of association between clinical history and radiographic features with microscopic pericoronal follicle examination for diagnosis of this hamartomatous lesion.

## Figures and Tables

**Table 1:** Review of the clinical and microscopic characteristics found in hyperplastic dental follicles of young patients.

Authors/year	Age/sex	Teeth affected*	Calcification	Enamel alteration	Single or multiple occurrence
van Heerden et al., 1990 [10]	14-year-old female	13 different teeth	Present	Amelogenesis imperfecta	Multiple

Gomez et al., 1998 [17]	15-year-old male	18, 17, 14, 13, 12, 23, 24, 27, 33, 43, and 48	Present	Unremarkable	Multiple

Onishi et al., 2003 [3]	5-year-10-month-old female	36 and 46	Present	Unremarkable	Not specified
	6-year-old female	36	Present	Unremarkable	Single

Walker et al., 2004 [4]	6-year-old female	85	Present	Unremarkable	Single

Feller et al., 2006 [9]	12 years old	17, 27, 37, 35, 33, 45, and 47	Present	Enamel displasia	Multiple

Nikitakis et al., 2006 [8]	14-year-old male	37 and 47	Present	Unremarkable	Not specified

Roquebert et al., 2008 [5]	10-year-old male	17, 16, 15, 14, 24, 25, 26, 27, 37, 36, 35, 34, 44, 45, 46, and 47	Present	Amelogenesis imperfecta	Multiple

Quintella et al., 2008 [6]	8-year-9-month-old female	36	Present	Unremarkable	Single

Sun et al., 2010 [24]	15-year-old male	13, 23, 33, and 43	Absent	Unremarkable	Multiple
	12-year-old female	13, 23, 33, and 43	Absent	Unremarkable	Multiple

Cho et al., 2011 [12]	11-year-old male	17, 13, 23, 27, 37, 36, and 47	Present	Unremarkable	Multiple
	14-year-old male	17, 13, 23, 27, 37, and 47	Present	Unremarkable	Multiple
	11-year-old male	17, 27, 37, and 47	Present	Unremarkable	Multiple
	15-year-old male	17, 15, 25, 27, 37, 35, and 47	Present	Unremarkable	Multiple

Jamshidi et al., 2013 [13]	19-year-old male	18, 17, 27, 28, 38, 37, 33, 43, 47, and 48	Present	Unremarkable	Multiple

O'Connell et al., 2014 [7]	7-year-old female	16, 26, 36, and 46	Present	Amelogenesis imperfecta	Multiple

Present case	14-year-old male	47	Present	Unremarkable	Single

*The ISO system by the World Health Organization was used for dental notation.
